# The Serine Acetyltransferase (*SAT*) Gene Family in Tea Plant (*Camellia sinensis*): Identification, Classification and Expression Analysis under Salt Stress

**DOI:** 10.3390/ijms25189794

**Published:** 2024-09-10

**Authors:** Leigang Wang, Dandan Liu, Xiaoyu Jiao, Qiong Wu, Wenjie Wang

**Affiliations:** Tea Research Institute, Anhui Academy of Agricultural Sciences, Hefei 230001, China; wangleigang@aaas.org.cn (L.W.); liudd521@aaas.org.cn (D.L.); jiaoxy@aaas.org.cn (X.J.)

**Keywords:** *Camellia sinensis*, *SAT* genes, qRT-PCR, prokaryotic expression

## Abstract

Cysteine plays a pivotal role in the sulfur metabolism network of plants, intimately influencing the conversion rate of organic sulfur and the plant’s capacity to withstand abiotic stresses. In tea plants, the serine acetyltransferase (*SAT*) genes emerge as a crucial regulator of cysteine metabolism, albeit with a notable lack of comprehensive research. Utilizing Hidden Markov Models, we identified seven *CssSATs* genes within the tea plant genome. The results of the bioinformatics analysis indicate that these genes exhibit an average molecular weight of 33.22 kD and cluster into three distinct groups. Regarding gene structure, *CssSAT1* stands out with ten exons, significantly more than its family members. In the promoter regions, cis-acting elements associated with environmental responsiveness and hormone induction predominate, accounting for 34.4% and 53.1%, respectively. Transcriptome data revealed intricate expression dynamics of *CssSATs* under various stress conditions (e.g., PEG, NaCl, Cold, MeJA) and their tissue-specific expression patterns in tea plants. Notably, qRT-PCR analysis indicated that under salt stress, *CssSAT1* and *CssSAT3* expression levels markedly increased, whereas *CssSAT2* displayed a downregulatory trend. Furthermore, we cloned *CssSAT1*-*CssSAT3* genes and constructed corresponding prokaryotic expression vectors. The resultant recombinant proteins, upon induction, significantly enhanced the NaCl tolerance of *Escherichia coli* BL21, suggesting the potential application of *CssSATs* in bolstering plant stress resistance. These findings have enriched our comprehension of the multifaceted roles played by *CssSATs* genes in stress tolerance mechanisms, laying a theoretical groundwork for future scientific endeavors and research pursuits.

## 1. Introduction

The delicate young shoots and leaves of *Camellia sinensis* serve as the primary ingredient for crafting high-quality teas. These teas are abundant in vital bioactive compounds such as tea polyphenols, caffeine, and amino acids, conferring notable health benefits when consumed [1,2,3]. The extensive cultivation of *C. sinensis* spans across Asia, Africa, the Americas, Oceania, and Europe, with the tea industry constituting a vital segment of local agriculture and the economy [4,5]. However, during its growth cycle, the tea plant inevitably confronts numerous abiotic stresses, notably soil salinization, drought, and late spring cold snaps, with drought and high salinity posing particularly formidable challenges [6]. These stresses not only disrupt the osmotic balance within plant cells but also significantly impair photosynthetic efficiency through the toxic effects of Na^+^ and Cl^−^ ions and the induction of oxidative stress mechanisms, ultimately hindering growth and yield [7,8]. Consequently, delving into the mechanisms underpinning *C. sinensis* resilience against abiotic stresses holds paramount significance for the sustainable development of the tea industry.

Serine acetyltransferase (SAT), a pivotal enzyme involved in cysteine biosynthesis, is ubiquitously distributed across various subcellular compartments within plant cells, including the cytoplasm, mitochondria, and chloroplasts [9,10]. The SAT proteins are composed of two crucial functional domains, SATase_N and Hexapep_C, both of which exhibit remarkable conservation across diverse plant species [11,12]. The *SAT* gene family, encoding serine acetyltransferases, plays a pivotal role in plant growth and development under abiotic stress conditions [9,13]. *SAT* mutants exhibit growth phenotypes consistent with reduced SAT activity, while quintuple mutants perish at the embryonic stage, underscoring the unique and indispensable role of *SAT* genes throughout the plant life cycle [14,15]. In *Arabidopsis thaliana*, the *SAT2;2* gene predominates in organic sulfur synthesis, whereas *SAT1;1*, *SAT3;1*, and *SAT3;2* are crucial for seed development [16]. Differential expression of *SAT* genes influences excessive nickel accumulation in *Thlaspi goesingense*, highlighting their significance in maintaining metal ion homeostasis [17]. Furthermore, overexpression of *SAT* genes significantly enhances the antioxidant capacity of *Arabidopsis*, particularly under sulfur deficiency and cadmium stress, where *SAT3;2* expression is prominently induced [18,19].

Under salt stress conditions, the redox balance within plant chloroplasts and mitochondria is disrupted, leading to the direct transfer of electrons to oxygen molecules, which gives rise to superoxide anion radicals (O_2_^−^). Subsequently, the accumulation of high concentrations of reactive oxygen species (ROS) triggers programmed cell death in plants [8,20]. To counteract this stress, cyclophilin CYP20-3 within the chloroplast stroma plays a pivotal role by facilitating the folding or assembly of SAT1 enzymes, thus participating in the biosynthetic pathway of cysteine, an effective response to oxidative stress [21]. As a crucial precursor for glutathione biosynthesis, increased cysteine levels contribute to reducing ROS accumulation, potentially enhancing salt tolerance in plants [8,22]. Experimental evidence has demonstrated that overexpressing *SAT* genes to enhance their enzymatic activity significantly boosted the content of cysteine, methionine, and glutathione in *Nicotiana tabacum*, while promoting the accumulation of zein in *Zea mays*. These findings present novel strategies for improving crop salt tolerance [23,24].

As the genomic research on tea plants continually deepens and progresses, molecular biology studies in this field have emerged as a pivotal direction within tea science research [25]. The *SAT* genes hold a crucial position in tea plants, indispensable for unraveling their metabolic mechanisms and enhancing stress resistance. Nonetheless, the current research endeavors pertaining to the *SAT* genes in tea plants are still inadequate, underscoring the urgency for comprehensive investigations into their gene structure, regulatory mechanisms of expression, and diverse biological functions. This study focuses on the identification and analysis of the *SAT* gene family at the whole-genome level in tea plants, with the objective of elucidating the variation in expression patterns of these genes across different tissues and environmental conditions. Particular emphasis is placed on investigating the expression changes of *SAT* genes under stress conditions such as salt stress, aiming to establish a theoretical foundation for a deeper understanding of how *SAT* genes respond to abiotic stresses. Ultimately, this research endeavors to contribute invaluable genetic resources for the genetic improvement and stress-tolerant breeding of tea plants.

## 2. Results

### 2.1. Genome-Wide Identification of SAT Genes in Tea Plant

**Figure 1:**
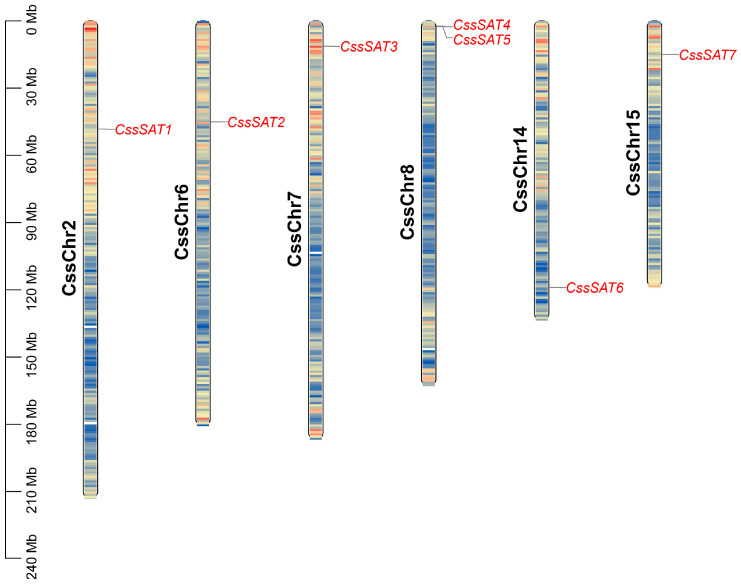
The distribution of *SAT* genes across the chromosomes in the tea plant. Red indicates a high density of genes on the chromosomes, while blue indicates a low density of genes.

During the data preprocessing phase, the contigs were excluded from the genome. Subsequently, a Hidden Markov Models (HMM) analysis was employed, with an E-value threshold set at E^−6^, to sieve out seven *SAT* genes from the genome (Supplementary Table S1). These genes are fairly evenly distributed across six chromosomes, with chromosomes 2, 6, 7, 14, and 15 each harboring one *SAT* gene, while chromosome 8 contains two (Figure 1). Following the physical order of genes on the chromosomes, they were sequentially named *CssSAT1* through *CssSAT7*.

Utilizing the online genomic analysis tool Softberry (http://www.softberry.com/, accessed on 31 May 2024), gene models were predicted based on the 5000 bp sequences flanking each of these genes. Incorrect annotations of adjacent P450 structures were removed, thereby rectifying the structural annotation of the *CssSAT3* gene. The *CssSATs* genes exhibit amino acid counts ranging from 270 to 354, with an average of 308; the molecular weights vary between 28.69 and 38.42 kD, averaging at 33.22 kD; and the isoelectric points span from 5.23 to 8.55, averaging 6.85, with a majority falling below 7.00, indicative of a high proportion of acidic amino acids (Table 1). It is predicted that these *CssSATs* genes are predominantly localized within the cytoplasm and chloroplasts.

### 2.2. Analysis of Multiple Sequence Alignment and Phylogenetic Relationships

**Figure 2:**
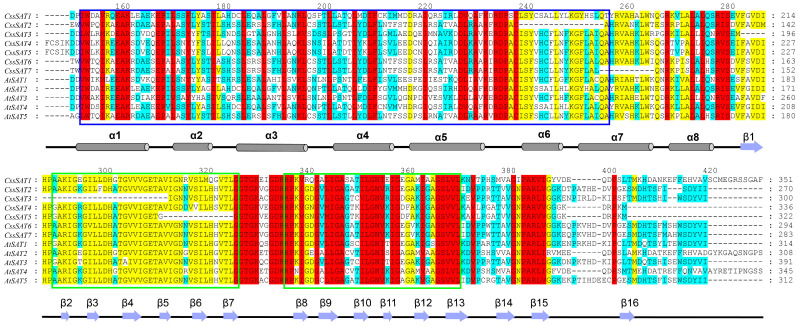
Multiple sequence alignment of amino acid sequences of *SAT* genes in *Camellia sinensis* and *Arabidopsis thaliana.* The red, yellow, and cyan colors signify a decreasing order of sequence similarity. The α-helix is indicated by a gray cylinder, and the β-fold is indicated by a purple arrow. The essential C-terminal Ile is indicated by a black pentagon. The SATase_N domain is labeled with a blue box, and the Hexapep_C domain is labeled with a green box.

The multiple sequence alignment results reveal a remarkable conservation of the N-terminal α-helix and C-terminal β-sheet structures in the tea plant and in *A. thaliana* (Figure 2). All aligned members encompass the N-terminal serine acetyltransferase domain (SATase_N, PF06426) and the unique C-terminal hexapeptide domain characteristic of bacterial transferases (Hexapep_C, PF00132). Notably, *CssSAT2* exhibits a truncation of 17 amino acid residues at its N-terminus compared to the other members, yet it maintains at least one Hexapep_C domain within its sequence. It is noteworthy that the C-terminal isoleucine (Ile) residue in *CssSAT1* and *AtSAT4* undergoes a mutation to valine (Val), accompanied by an extension at the C-terminal tail. Furthermore, *CssSAT4* and *CssSAT5* display prominent deletions in their C-terminal tails when compared to other SAT proteins, suggesting unique structural or functional adaptations in these variants.

**Figure 3:**
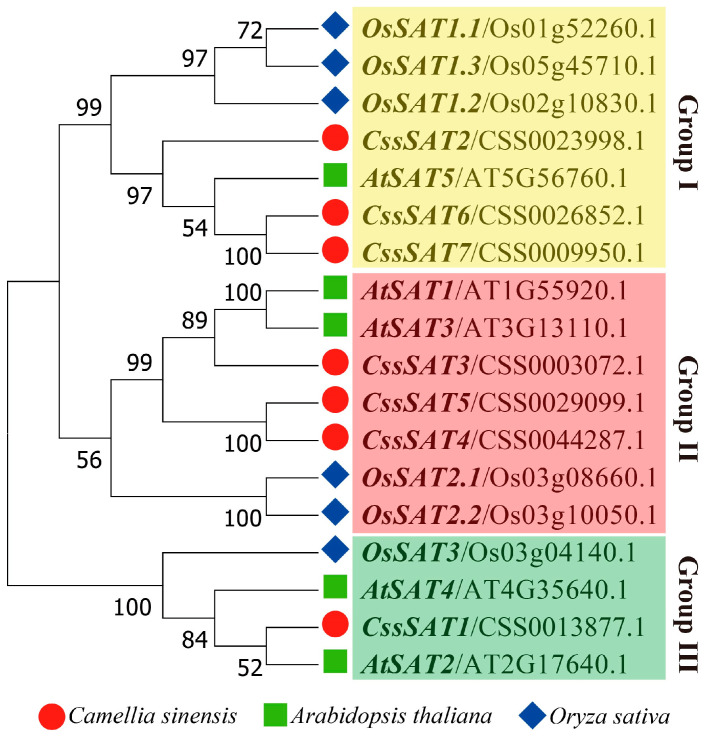
Phylogenetic relationship between *Camellia sinensis* and the *SAT* genes of *Arabidopsis thaliana* and *Oryza sativa*. The phylogenetic tree was constructed using the neighbor-joining (NJ) method in MEGA v6.06 software, incorporating 1000 bootstrap replicates.

The evolutionary trees of tea plant, *A. thaliana*, and rice, constructed using the neighbor-joining (NJ) method, categorized the 18 *SAT* sequences from these three species into three distinct groups labeled Group I to III (Figure 3). Notably, tea plant exhibits a balanced representation in Groups I and II, with three members in each, whereas Group III comprises solely one member. Delving deeper, intragroup subdivisions are evident within Groups I and II. *CssSAT6*, *CssSAT7*, as well as *CssSAT4* and *CssSAT5*, form tight gene clusters, categorizing them into a common subclass. Additionally, *AtSAT1* and *AtSAT3* within Group II, along with *OsSAT2.1* and *OsSAT2.2*, exhibit a high degree of homology, further underscoring their close evolutionary or functional relationships.

### 2.3. Analysis of the Conserved Structure and Genetic Architecture of CssSATs

**Figure 4:**
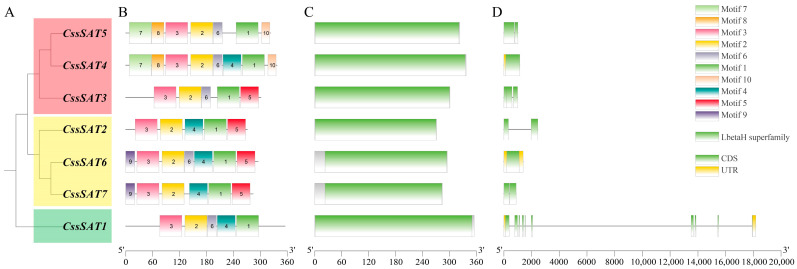
The depiction encompasses (**A**) the phylogenetic tree, (**B**) conserved motifs, (**C**) conserved domains, as well as (**D**) exons and introns of *CssSATs* genes.

The evolutionary tree constructed via Maximum Likelihood (ML) methodology distinctly segregates *CssSAT* genes into three distinct categories: *CssSAT1* stands alone as a separate group; *CssSAT2*, *CssSAT6*, and *CssSAT7* coalesce into the second group; and *CssSAT3*, *CssSAT4*, and *CssSAT5* form the third cluster (Figure 4A). Notably, the close phylogenetic relationships between *CssSAT6* and *CssSAT7*, as well as between *CssSAT4* and *CssSAT5*, corroborate previous observations. Furthermore, Motif prediction illuminates the underlying characteristics of potential conserved domains. Among the ten identified motifs, motifs 1 to 3 exhibit a universal presence across all genes, indicative of their high conservation. Conversely, motifs 7, 8, and 10 are specifically found in *CssSAT4* and *CssSAT5*, while motif 9 is uniquely associated with *CssSAT6* and *CssSAT7* (Figure 4B). Additionally, the proteins encoded by these genes uniformly harbor the left-handed parallel β-helix (LbetaH) domain, a structural feature often associated with acyltransferase activity (Figure 4C). Regarding gene structure, *CssSAT1* boasts the highest number of exons with ten; *CssSAT3* contains three exons; *CssSAT2*, *CssSAT5*, and *CssSAT7* each possess two exons; and both *CssSAT4* and *CssSAT6* harbor only a single exon (Figure 4D). These findings offer insights into the structural and functional diversity within the *CssSAT* gene family.

### 2.4. Analysis of Cis-Acting Elements in the Promoter Region of the CssSATs Genes

**Figure 5:**
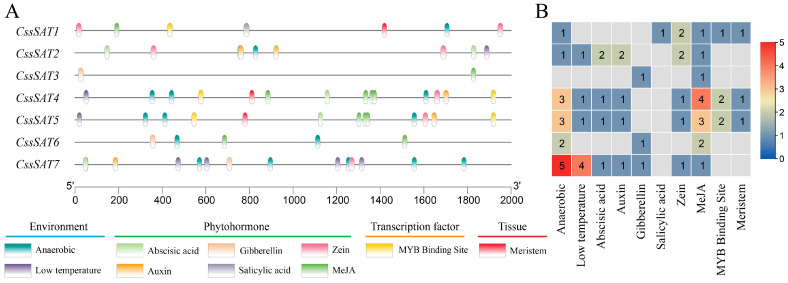
Illustration of cis-acting elements within the promoter region of *CssSATs* genes. (**A**) Representation of the distribution of different types of cis-acting elements. (**B**) A heatmap depicting the abundance of cis-acting elements.

In our investigation of the promoters of the *CssSATs* genes in tea plant, we not only validated the presence of the ubiquitous TATA-box and CAAT-box elements but also identified a repertoire of functional specificity elements intimately associated with environmental adaptability, hormonal responses, transcriptional factor regulation, and tissue-specific development (Figure 5). Notably, environmental response and hormone-inducible elements emerged as dominant players, accounting for 34.4% (22/64) and 53.1% (34/64), respectively, underscoring their pivotal roles in modulating gene expression. The quantity and distribution of these elements varied significantly among genes, ranging from 2 to 14, averaging 9 elements per gene (Supplementary Table S2).

Remarkably, all genes featured methyl jasmonate-associated elements, with *CssSAT4* and *CssSAT5* exhibiting particularly prominent enrichment, suggesting a pervasive involvement of methyl jasmonate signaling in tea plants. Furthermore, *CssSAT1* uniquely possessed salicylic acid-responsive elements, while *CssSAT4*, *CssSAT5*, and *CssSAT7* shared anaerobic stress-responsive elements, with three, three and five, respectively, hinting at their potential regulatory functions under specific environmental cues. Of particular interest, *CssSAT7* harbors four low-temperature response elements, portending its crucial function in the tea plant’s response mechanism to chilling stress. This finding highlights the intricate interplay between genetic elements and environmental stimuli, underscoring the complexity and sophistication of regulatory networks governing tea plant development and adaptation.

### 2.5. Analysis of Expression Profiles of the CssSATs Genes under Different Stress Conditions

**Figure 6:**
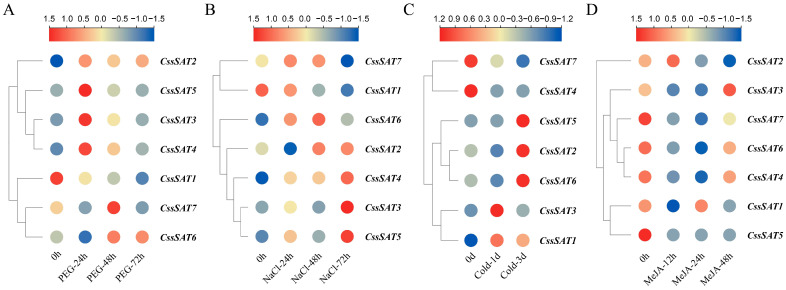
Transcriptional expression profiles of *CssSATs* genes in response to diverse stress conditions. (**A**) PEG treatment. (**B**) NaCl treatment. (**C**) Cold stress. (**D**) MeJA treatment.

Under PEG treatment, the expression patterns of *CssSATs* genes in tea plant exhibited diverse trends (Figure 6A). Specifically, *CssSAT1* displayed negative regulation, with its expression levels continuously declining over 72 h, whereas *CssSAT2* showed an ascending trend. Additionally, *CssSAT3*, *CssSAT4*, and *CssSAT5* peaked in expression at 24 h, whereas *CssSAT6* and *CssSAT7* reached their maxima at 48 h.

Under NaCl treatment, *CssSAT1* followed a similar negative regulatory pattern as observed under PEG treatment. Conversely, *CssSAT3*, *CssSAT4*, and *CssSAT5* peaked in expression at 72 h, while *CssSAT6* and *CssSAT7* initially increased at 24 and 48 h before declining at 72 h (Figure 6B).

Under cold treatment, the expression of *CssSATs* genes in tea plant demonstrated three distinct trends: *CssSAT1* and *CssSAT3* peaked at 1 d and subsequently decreased slightly by 3 d; *CssSAT2*, *CssSAT5*, and *CssSAT6* showed elevated expression levels at 3 d; and *CssSAT4* and *CssSAT7*, however, exhibited a declining trend (Figure 6C).

In response to MeJA treatment, the overall expression patterns of *CssSATs* genes were predominantly negatively regulated. Specifically, *CssSAT2* displayed an initial increase in expression at 12 h followed by a decrease. *CssSAT3*, *CssSAT4*, *CssSAT6*, and *CssSAT7* showed decreased expression at 12 and 24 h but slightly increased at 48 h (Figure 6D).

### 2.6. Analysis of the Expression Profile of CssSATs Genes in Diverse Tissues of Tea Plant

**Figure 7:**
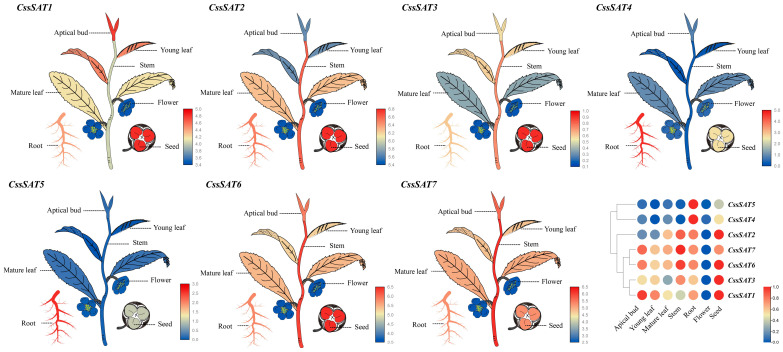
Transcriptional expression profiles of *CssSATs* genes across diverse tissues. Diagram manually prepared using Adobe Illustrator v22.1 and exported in SVG format. SVG imported into the eFP browser for gene expression data visualization. Data transformed using base-2 logarithmic formula: log(TPM + 1) for enhanced sensitivity and clarity. Low expression in blue, high expression in red hues.

The expression patterns of *CssSATs* genes exhibit notable variations across the seven tissue types of tea plant (Figure 7). Specifically, *CssSAT1* displays a declining trend in its expression levels from buds, young leaves, mature leaves, to stems, whereas *CssSAT2* follows the opposite trend. Notably, *CssSAT4* and *CssSAT5* exhibit highly specific patterns exclusively in roots. Furthermore, the expression values of *CssSAT1*, *CssSAT2*, *CssSAT6*, and *CssSAT7* generally surpass those of *CssSAT3*, *CssSAT4*, and *CssSAT5*. Additionally, there are similarities in the expression patterns between *CssSAT3* and *CssSAT1*, as well as between *CssSAT7* and *CssSAT6*. It is noteworthy that *CssSATs* genes tend to be highly expressed in roots and seeds, whereas their expression in flowers is relatively lower. This analysis suggests potential functional differences among various *CssSATs* gene members, with some genes being specific to certain tissues or developmental stages.

### 2.7. Analysis of qRT-PCR Results for the CssSATs Genes

**Figure 8:**
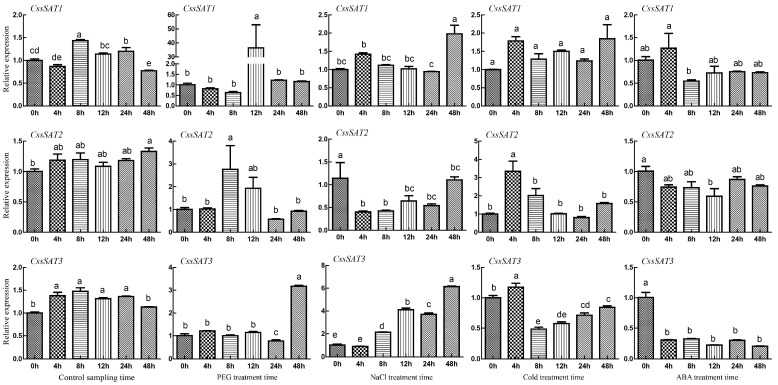
The relative expression levels of selected *CssSATs* genes under diverse stress conditions, as determined by qRT-PCR. The error bars represent the standard deviation derived from three independent biological replicates, where distinct lowercase letters signify statistically significant differences observed at various time points within the same treatment (*p* < 0.05).

This study delved into the expression dynamics of three genes, *CssSAT1*, *CssSAT2*, and *CssSAT3*, over a 48 h period using qRT-PCR technology under simulated drought, salt, cold stress, and ABA treatment conditions (Figure 8). The results from the control samples revealed that the expression patterns of *CssSATs* genes exhibited minimal temporal fluctuations within 48 h, indicating a relatively stable expression profile.

Under drought stress, *CssSAT1* exhibited a marked increase in expression at 12 h, suggesting its potential heightened sensitivity to drought at this specific time point. Conversely, *CssSAT2* demonstrated a significant upregulation as early as 8 h, indicating a more rapid response. Meanwhile, *CssSAT3* showed a substantial elevation in expression levels at 48 h, implying its potential role in long-term drought adaptation. Under salt stress conditions, *CssSAT1* exhibited a notable upregulation in expression levels at 48 h, suggesting its potential involvement in the later adaptive mechanisms to salt stress. Conversely, *CssSAT2* showed a downregulation in expression, which may be associated with a negative regulatory mechanism under salt stress. Meanwhile, *CssSAT3* demonstrated a sustained increase in expression, reaching a considerable level at 48 h, likely indicating its crucial role in the synthesis of substances related to salt stress tolerance.

In response to low temperature stress, the expression level of *CssSAT1* remained largely unchanged, suggesting it may not be directly involved in the low temperature response mechanism. Conversely, *CssSAT2* and *CssSAT3* exhibited a transient upregulation followed by a decrease in expression after 4 h, a dynamic pattern that likely reflects their initial response to the stress and subsequent adaptation process. During ABA treatment, the expression of *CssSAT1*, *CssSAT2*, and *CssSAT3* all displayed a downregulation trend, with *CssSAT3* showing the most pronounced effect by stabilizing at a significantly lower level. This observation implies that ABA may participate in other stress response pathways by negatively regulating the expression of these genes.

### 2.8. Analysis of Prokaryotic Expression of the CssSATs Genes

**Figure 9:**
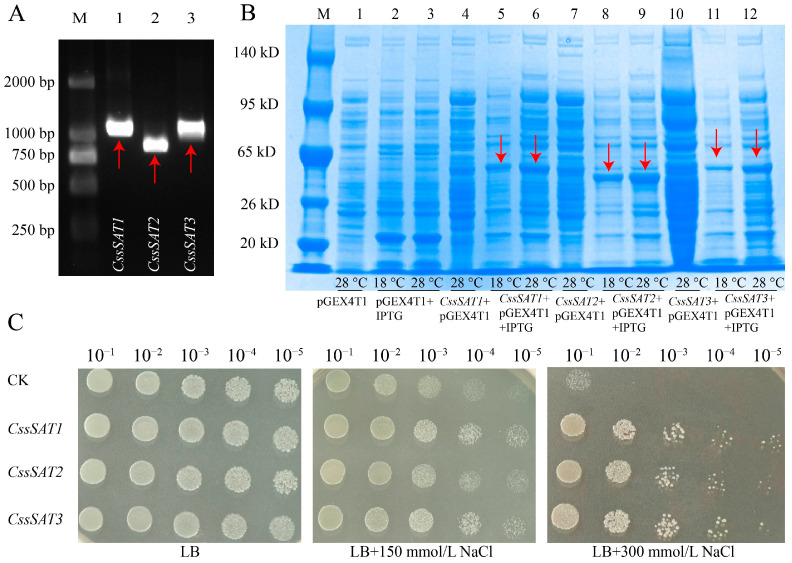
Prokaryotic expression of *CssSATs* genes. Red arrows indicate target genes or proteins. (**A**) Cloning of *CssSATs*. (**B**) Expression of *CssSATs* proteins in *E. coli*. (**C**) Growth profile of *CssSATs* recombinant *E. coli* cultivated in media with varying concentrations of NaCl.

In this study, we successfully amplified and cloned the CDS sequences of *CssSAT1*, *CssSAT2* and *CssSAT3* genes from the cDNA of the ‘Shuchazao’ tea plant (Figure 9A). Subsequently, we constructed the corresponding prokaryotic expression vectors, namely, pGEX-4T-1-CssSAT1, pGEX-4T-1-CssSAT2, and pGEX-4T-1-CssSAT3, which were then transformed into *Escherichia coli* BL21 (DE3) for expression. Upon IPTG induction, SDS-PAGE analysis revealed efficient expression of *CssSAT1*, *CssSAT2* and *CssSAT3* fusion proteins under both 18 °C and 28 °C conditions, with a notable increase in protein concentration as the temperature rose (Figure 9B).

To further investigate, we assessed the response of *E. coli* expressing the pGEX-4T-1-CssSATs fusion proteins to NaCl stress through spot assays. Our results indicated that, in standard LB medium, the growth patterns of the recombinant strains (BL21 + pGEX-4T-1-CssSATs) were comparable to those of the control strain (BL21 + pGEX-4T-1). However, when grown on media containing 150 mmol/L NaCl, the recombinant strains exhibited a significant growth advantage over the control, even after a 10^4-fold dilution. Moreover, under more severe NaCl stress conditions (300 mmol/L NaCl), all *CssSATs* recombinant strains displayed enhanced tolerance compared to the control (Figure 9C). These findings underscore the ability of *CssSATs* recombinant proteins to markedly bolster the resistance of *E. coli* BL21 cells against NaCl stress.

## 3. Discussion

The *SAT* genes, being a pivotal encoding factor in the pathway of sulfur assimilation into cysteine, are ubiquitously present across a diverse array of organisms, encompassing but not limited to bacteria, fungi, and plants [26]. In the tea plant genome, we have identified an exceptional number *CssSATs* genes (seven), surpassing the numbers observed in other species, such as *Solanum lycopersicum* (four), *Allium sativum* (five), and *A. thaliana* (five), among others [27,28,29]. This finding hints at a potentially more intricate evolutionary trajectory undertaken by the tea plant within the *CssSATs* gene family. Notably, not only does the tea plant boast a substantial quantity of *CssSATs* genes, but they are also widely dispersed across multiple chromosomes, a distribution pattern that could potentially enhance genetic diversity and stability.

Furthermore, the recent paleopolyploidy event in the tea plant genome has significantly contributed to genome reorganization and expansion, likely profoundly influencing the distribution and abundance of the *CssSATs* gene family [30]. Notably, intron insertion, a pivotal mechanism underpinning gene family expansion and differentiation, stands out particularly in the *CssSAT5* and *CssSAT7* genes of the tea plant. These genetic and evolutionary variations may have led to diversification in the expression patterns, functional properties, and regulatory mechanisms of *CssSATs* genes, ultimately endowing tea plants with enhanced environmental adaptability and a rich genetic repertoire.

The C-terminal architecture of SAT proteins exhibits a remarkable degree of conservation, with mutations at crucial amino acid positions leading to a substantial decrease in their enzymatic activity [31,32]. Notably, the Ile residue within the C-terminus of SAT proteins serves as a pivotal “anchor” for interaction with O-acetyl serine (thiol) lyase (OASTL) proteins, not only being intimately associated with the enzymatic active site but also playing a central role in the feedback inhibition mechanism involving cysteine [33,34,35]. The SAT enzyme family in tea plants demonstrates significant diversity, notably through mutations or deletions of the Ile residue in the C-terminus of CssSAT1, CssSAT4, and CssSAT5 proteins, a feature that likely influences their mutual recognition and functional interplay with the CssOASTL protein. Consequently, future research endeavors should prioritize an in-depth investigation into the interaction mechanisms between these SAT proteins and OASTL, as well as how they collaboratively respond to abiotic stresses encountered by tea plants.

The research demonstrates that tomato chloroplast SAT activity undergoes a notable increase 24 h post-salt stress exposure, with *SlSAT2;2* transcript levels being significantly upregulated, thereby identifying this gene as one of the primary responsive genes to salt stress [28]. In the case of barley (*Hordeum vulgare*), exogenous cysteine significantly mitigates the accumulation of O_2_^−^ and H_2_O_2_ in root and embryo tissues, playing a crucial role in maintaining cellular redox homeostasis [36]. Our qRT-PCR analysis unveils the differential responsiveness of the *CssSATs* gene family to salt stress, with *CssSAT3* gene expression consistently and markedly upregulated over extended periods of stress. Furthermore, the introduction of *CssSAT1*, *CssSAT2*, and *CssSAT3* genes into *E*. *coli* via a prokaryotic expression system significantly enhances the host cells’ tolerance to salt stress. Similarly, rice *OsSAT1;1*, *OsSAT1;2*, and *OsSAT1;3* genes are upregulated across various stages of salt stress, and some *OsSAT* gene expressions are induced by jasmonic acid (JA) [37]. JA and MeJA, in concert with multiple plant hormones, orchestrate plant responses to salt stress within intricate signaling networks [38,39]. Under salt stress, JA and ABA concentrations surge, and exogenous JA treatment can decrease Na+ absorption in maize roots, improve Na+ exclusion in stems, and potentially inhibit leaf growth in salt-sensitive genotypes [40]. Additionally, MeJA effectively alleviates salt stress-induced oxidative stress in wheat (*Triticum aestivum*) seedlings by elevating antioxidant enzyme activities and compound concentrations [41]. In this study, MeJA treatment led to a transient increase in *CssSAT2* gene expression at 12 h, while other *CssSAT* genes exhibited fluctuating responses, likely attributed to the complexity of isozyme expression within the gene family and the multifaceted regulation of enzyme activity.

Drought conditions markedly enhance the excessive generation of ROS within plant cells, subsequently triggering lipid peroxidation reactions that significantly damage cellular membranes [42,43]. Under drought stress, the levels of ROS within maize leaves significantly upregulate the transcription of key isoform genes, including *SAT1;1*, *SAT2;1*, and *SAT2;2*, triggering a mechanism that subsequently activates endogenous SAT enzyme activity [44]. This activation accelerates the assimilation process of sulfate and enhances the production of antioxidants, ultimately facilitating maize plants to effectively cope with drought conditions [44]. Similarly, our study reveals that under PEG-simulated drought stress, the expression of CssSAT1 gene soars, peaking at 36.5-fold the control levels within 12 h, underscoring its pivotal role in drought response.

Remarkably, in the case of ABA, a pivotal regulator in drought stress, the transcription of the biosynthetic and metabolic genes, *ABA3* and *NCED3*, is significantly modulated by sulfur supply, indicating a close interplay between sulfur metabolism and ABA biosynthesis [45]. Furthermore, drought stress inhibits the expression of the ABA catabolic gene *ABA8ox1*, while exogenous sulfide treatment effectively improves ABA accumulation under drought stress by promoting ABA biosynthesis, thereby enhancing plant drought tolerance [46]. Our investigation delves deeper to uncover that upon exogenous ABA application, the expression of *CssSAT1*, *CssSAT2*, and *CssSAT3* genes exhibits a downward trend. This suggests that these genes might indirectly affect ABA biosynthesis and metabolism through negatively regulating tea plant *SAT* gene expression, thereby participating in adaptive responses to drought stress.

Under H_2_O_2_-induced oxidative stress conditions, in vitro cellular experiments have demonstrated that the NV14 peptide derived from the *ApSAT* gene notably modulates the expression of key glutathione metabolic genes, including glutathione peroxidase (GPx), glutathione S-transferase (GST), and γ-glutamylcysteine ligase (GCL), effectively diminishing intracellular ROS levels and thereby positively regulating the cellular antioxidant defense system [47]. Furthermore, the engineering and overexpression of a feedback-insensitive isoform of tobacco SAT enzyme, *NtSAT4*, not only facilitated the accumulation of cysteine and GSH, alleviating plant wilting and pigment alterations triggered by oxidative stress, but also significantly bolstered plant tolerance against oxidative challenges posed by hydrogen peroxide and heavy metal cadmium (Cd) [48,49]. A separate investigation revealed that enhanced activity of the wild-type soybean SAT1 enzyme under light and oxidative stress impacted thiol accumulation and photosynthetic pigment content within chloroplasts, underscoring the vital and distinct physiological regulatory role of plant *SAT* genes in responding to oxidative stress [21,50].

The future prospects of our study are promising and multifaceted. We plan to adopt a multi-omics approach, integrating transcriptomics, proteomics, and metabolomics technologies to comprehensively elucidate expression patterns, construct protein–protein interaction networks, and dissect metabolic pathways. Building upon this solid foundation, we aim to further investigate the regulatory mechanisms of *SAT* genes under salt stress conditions, with a particular emphasis on uncovering their potential applications in tea plant breeding. Our ultimate aspiration is to develop novel tea plant varieties that not only exhibit superior quality traits but also possess heightened stress resilience, thereby contributing to the sustainability and productivity of tea cultivation.

## 4. Materials and Methods

### 4.1. Experimental Materials

The experimental study utilized uniformly vigorous and healthy annual ‘Shuchazao’ tea plants (*C. sinensis* var. *sinensis*), meticulously selected from the tea plant nursery of the Tea Research Institute, Anhui Academy of Agricultural Sciences, located in Huangshan city, China. Four distinct stress treatment conditions were devised for the study. Specifically, drought stress, simulated using a 15% polyethylene glycol 6000 (PEG 6000) solution (*w*/*v*); salt stress, mimicked with a 200 mmol/L sodium chloride (NaCl) solution; Abscisic acid (ABA) treatment, applied as a 100 μmol/L ABA solution; and low-temperature stress, replicated in an environment maintained at 4 ± 1 °C.

Concurrently, a control group was established, consisting of tea plants grown under normal conditions at 24 °C. Sampling was conducted at 0, 4, 8, 12, 24, and 48 h, with the second fully matured leaf from each treated plant being harvested promptly and immediately fixed in liquid nitrogen. Each treatment was replicated three times to ensure the reliability and statistical significance of the experimental outcomes.

### 4.2. Screening and Sequence Analysis of CssSATs Genes

The Hidden Markov Model (HMM) PF06426 for *SAT* genes was downloaded from the Pfam database (http://pfam.xfam.org/, accessed on 30 May 2024). Subsequently, the genomic data of the ‘Shuchazao’ tea plant was obtained from the Tea Plant Information Archive 2 (TPIA2) (http://tpia.teaplants.cn/, accessed on 30 May 2024) [51]. Utilizing the bioinformatics platform software TBtools-II v2.098 [52], we conducted a search based on the HMM to retrieve the *CssSATs* sequences in the tea plant. To analyze the distribution of these genes, we employed the Gene Density Profile tool within TBtools, leveraging GFF3 annotation files to visualize the gene density across chromosomes, providing a more intuitive representation of the gene locations.

To gain further insights into the physicochemical properties of these proteins, we utilized the ProtParam tool (https://web.expasy.org/protparam/, accessed on 10 June 2024) to calculate key parameters such as amino acid count, protein molecular weight, and isoelectric point. Additionally, subcellular localization predictions for the proteins were performed using WoLF PSORT (https://www.genscript.com/wolf-psort.html, accessed on 10 June 2024), enhancing our understanding of their cellular contexts.

### 4.3. Alignment and Phylogenetic Analysis of CssSATs Sequences

We downloaded the genomic data of *Arabidopsis thaliana* and *Oryza sativa* from the Phytozome database (https://phytozome-next.jgi.doe.gov/, accessed on 20 June 2024) and retrieved the corresponding sequence information based on published *SAT* gene identifiers. Subsequently, we aligned the acquired multiple sequences using the MEGA v6.06 [53] software, followed by refining and enhancing the alignment results with the aid of GeneDoc. To delve into structural characteristics, we employed the Phyre2 protein secondary structure prediction platform (http://www.sbg.bio.ic.ac.uk/phyre2/, accessed on 20 June 2024) to analyze features such as alpha-helices and beta-sheets.

Furthermore, we constructed a phylogenetic tree of *SAT* genes among *C. sinensis*, *A. thaliana*, and *O. sativa* using the neighbor-joining (NJ) method implemented in MEGA v6.06. This phylogenetic tree was exported in the Newick format for subsequent analysis. Finally, we leveraged the vector graphics software Adobe Illustrator v22.1 to visually represent and aesthetically enhance the phylogenetic tree, producing a clear and informative illustration.

### 4.4. Analysis of the Structure and Conserved Motifs of CssSATs Genes

To identify ten motifs within the SAT proteins of tea plant, we employed the Simple MEME Wrapper tool within TBtools. Subsequently, we predicted the conserved domains of these SAT proteins using the Conserved Domain Database (CDD) (https://www.ncbi.nlm.nih.gov/cdd/, accessed on 21 June 2024). Furthermore, we analyzed the exon-intron structure of *SAT* genes based on the GFF3 file of tea plant. To visually represent the evolutionary tree, motifs, domains, and gene structures of *SAT* genes, we leveraged the advanced Gene Structure View tool within TBtools, providing a comprehensive visualization of these features.

### 4.5. Analysis of Cis-Acting Elements within the Promoter Region of CssSATs Genes

To predict regulatory elements, we extracted the 2000 bp sequence upstream of the ATG start codon of the *SAT* genes from the tea plant genome. Subsequently, these sequences were submitted to the PlantCARE database (https://bioinformatics.psb.ugent.be/webtools/plantcare/html/, accessed on 22 June 2024) for analysis. Following the collation and analysis of the predicted outcomes, we utilized the Simple BioSequence Viewer and HeatMap tools within TBtools to visualize the data, facilitating comprehensive interpretation.

### 4.6. Analysis of the Transcriptional Expression of CssSATs Genes

To acquire transcriptome data of the ‘Shuchazao’ tea plant across various tissues [54], we downloaded the necessary information from TPIA2, along with transcriptome expression data under PEG, NaCl treatment [55], cold stress [56], and methyl jasmonate (MeJA) exposure [57] conditions. Subsequently, we searched for TPM values of the *SAT* genes (Supplementary Tables S3 and S4) and visualized these data using the HeatMap tool in TBtools. To elucidate expression trends more clearly, we applied a log2 transformation and intra-gene normalization to the data. Furthermore, we leveraged TBtools’ eFP browser to generate illustrative heatmap diagrams representing different tissues.

To delve deeper into the expression pattern of the *SAT* genes, high-quality total RNA was extracted from the samples mentioned in Section 4.1. This RNA was then reverse-transcribed into cDNA. Utilizing the Primer3 website (https://primer3.ut.ee/, accessed on 8 July 2024), we designed specific primers for qRT-PCR experiments (Supplementary Table S5). To ensure the reliability of our findings, we selected the universally recognized *GAPDH* gene in tea plant as an internal control (Supplementary Table S6). The relative expression levels of the *SAT* genes were calculated using the 2^−ΔΔCt^ method.

### 4.7. Analysis of the Prokaryotic Expression of CssSATs Genes and Salt Tolerance of the Recombinant Bacterial Strain

To amplify the coding sequence (CDS) of the *SAT* genes from tea plant utilizing PCR technology, the amplified products were subsequently ligated into the pGEX-4T-1 vector and transformed into *Escherichia coli* BL21 cells, thereby constructing a prokaryotic expression vector for the *CssSATs* genes. Positive clones were screened through colony PCR and sequencing validation, followed by expanded culturing of the confirmed strains. Once the OD600 of the bacterial suspension reached approximately 0.8, IPTG was added to a final concentration of 0.5 mmol/L, and protein expression was induced at 18 °C, 28 °C, and 37 °C for 8 h each in a shaker incubator. The resulting proteins were extracted and analyzed via SDS-PAGE to optimize induction conditions for the recombinant BL21 strain.

To further investigate the salt tolerance of the *CssSATs* recombinant proteins, bacterial suspensions grown in LB broth containing ampicillin (OD600 adjusted to 0.6) were subjected to gradient dilutions (10^1^, 10^2^, 10^3^, 10^4^, 10^5^ fold). These diluted suspensions were then plated onto LB agar containing varying concentrations of NaCl (0, 150, 300 mmol/L) and 50 mg/L ampicillin. As a control, BL21 strains harboring the empty pGEX-4T-1 vector were similarly treated. All strains were incubated overnight at 37 °C, and their growth patterns were subsequently recorded and analyzed.

## 5. Conclusions

In this study, we have identified seven *CssSATs* genes within the tea plant genome and categorized them into three distinct groups through phylogenetic tree analysis, with each group exhibiting highly similar gene structural features and conserved motifs. Notably, the promoter regions of these genes are enriched with diverse cis-acting elements intimately associated with environmental stress responses and hormone signaling pathways, underscoring their pivotal roles in plant adaptation to changing environments. Transcriptome profiling further illuminated the robust responsiveness of the *CssSATs* gene family to PEG, NaCl, cold, and MeJA, demonstrating their extensive potential in regulating stress tolerance. Additionally, qRT-PCR data revealed a marked upregulation of *CssSAT3* expression under salt stress conditions, emphasizing the pivotal function of *CssSAT3* in the salt stress response. Notably, the recombinant *CssSATs* proteins significantly enhanced the survival of *E. coli* under high salt conditions, further illuminating the potential application of the *CssSATs* genes in enhancing plant stress resistance.

## Figures and Tables

**Table 1:** Basic information of *SAT* genes identified in *Camellia sinensis*.

Gene Name	Gene ID	Chromosomal Localization	AA ^1^	MW ^2^ (kD)	pI ^3^	Subcellular Localization
*CssSAT1*	CSS0013877.1	Chr2: 48272763-48290939	354	38.42	5.23	Cytoplasm
*CssSAT2*	CSS0023998.1	Chr6: 45069503-45071954	270	28.69	6.25	Cytoplasm
*CssSAT3*	CSS0003072.1	Chr7: 11430067-11431056	300	32.90	6.85	Chloroplast
*CssSAT4*	CSS0044287.1	Chr8: 2394885-2396035	336	36.39	8.07	Cytoplasm
*CssSAT5*	CSS0029099.1	Chr8: 2527843-2528854	322	34.90	8.55	Cytoplasm
*CssSAT6*	CSS0026852.1	Chr14: 118914206-118915594	294	31.28	6.70	Cytoplasm
*CssSAT7*	CSS0009950.1	Chr15: 15007141-15008025	283	29.98	6.29	Cytoplasm

^1^ AA, number of amino acid; ^2^ MW, molecular weight; ^3^ pI, isoelectric point.

## Data Availability

Data are contained within the article and Supplementary Materials.

## Supplementary Materials

The supporting information can be downloaded at: https://www.mdpi.com/article/10.3390/ijms25189794/s1.
